# Primary headaches during the COVID-19 lockdown in Germany: analysis of data from 2325 patients using an electronic headache diary

**DOI:** 10.1186/s10194-021-01273-z

**Published:** 2021-06-22

**Authors:** Bianca Raffaelli, Jasper Mecklenburg, Simon Scholler, Lucas Hendrik Overeem, Ana Sofia Oliveira Gonçalves, Uwe Reuter, Lars Neeb

**Affiliations:** 1 Department of Neurology, Charité – Universitätsmedizin Berlin, Charitéplatz 1, 10117 Berlin, Germany; 2 Newsenselab GmbH, Blücherstraße 22, 10961 Berlin, Germany; 3 Institute of Public Health, Charité – Universitätsmedizin Berlin, Charitéplatz 1, 10117 Berlin, Germany

**Keywords:** COVID-19, Lockdown, Primary headache, Migraine

## Abstract

**Background:**

Lockdown measures due to the COVID-19 pandemic have led to lifestyle changes, which in turn may have an impact on the course of headache disorders. We aimed to assess changes in primary headache characteristics and lifestyle factors during the COVID-19 lockdown in Germany using digital documentation in the mobile application (app) M-sense.

**Main body:**

We analyzed data of smartphone users, who entered daily data in the app in the 28-day period before lockdown (baseline) and in the first 28 days of lockdown (observation period). This analysis included the change of monthly headache days (MHD) in the observation period compared to baseline. We also assessed changes in monthly migraine days (MMD), the use of acute medication, and pain intensity. In addition, we looked into the changes in sleep duration, sleep quality, energy level, mood, stress, and activity level. Outcomes were compared using paired t-tests.

The analysis included data from 2325 app users. They reported 7.01 ± SD 5.64 MHD during baseline and 6.89 ± 5.47 MHD during lockdown without significant changes (*p* > 0.999). MMD, headache and migraine intensity neither showed any significant changes. Days with acute medication use were reduced from 4.50 ± 3.88 in the baseline to 4.27 ± 3.81 in the observation period (*p* < 0.001). The app users reported reduced stress levels, longer sleep duration, reduced activity levels, along with a better mood, and an improved energy level during the first lockdown month (*p* ≤ 0.001).

In an extension analysis of users who continued to use M-sense every day for 3 months after initiation of lockdown, we compared the baseline and the subsequent months using repeated-measures ANOVA. In these 539 users, headache frequency did not change significantly neither (6.11 ± 5.10 MHD before lockdown vs. 6.07 ± 5.17 MHD in the third lockdown month, *p* = 0.688 in the ANOVA). Migraine frequency, headache and migraine intensity, and acute medication use were also not different during the entire observation period.

**Conclusion:**

Despite slight changes in factors that contribute to the generation of headache, COVID-19-related lockdown measures did not seem to be associated with primary headache frequency and intensity over the course of 3 months.

## Introduction

Patients with primary headache disorders perceive external factors such as stress, sleep, and changes of everyday routine as common trigger of their headache attacks [1]. Especially patients with migraine are susceptible to both external and endogenous triggers, which might be related to changes in neuronal excitability networks [2]. However, also patients with other primary headache disorders such as tension-type headache (TTH) usually report one or more precipitating factors, most commonly related to changes in stress levels or sleep patterns [1]. Therefore, lockdown measures due to the coronavirus disease 2019 (COVID-19), which changed daily life dramatically, could have an influence on the course of primary headaches.

**Fig. 1:**
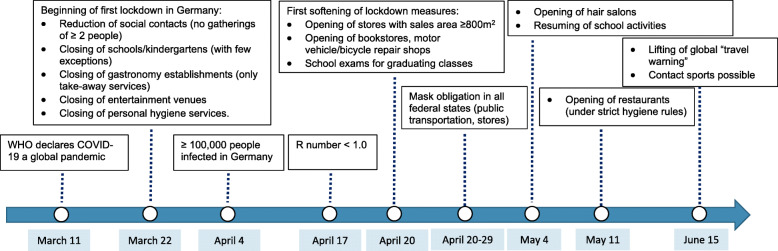
Timeline of the first lockdown in Germany in 2020

On March 11, 2020, the World Health Organization (WHO) declared COVID-19 a global pandemic, and on March 22, the German government imposed strict social distancing measures on public life [3]. The restrictions included a ban on public gatherings of more than two people, the closing of restaurants, bars, culture and sports venues, personal hygiene services, shops, schools, and kindergartens. Workers in “non-essential” professions were encouraged to work from home. From April 20, the lockdown measures in Germany were successively softened [4], with small differences in the sixteen federal states, but restrictions in numerous public and private activities remained at least until June, as shown in Fig. 1 [5].

Studies on mental health during the COVID-19 pandemic suggest a high psychosocial impact of such measures with a negative effect on quality of life, beginning in the first lockdown weeks [6, 7]. Patients with chronic diseases, including headache disorders, had to face cancellations of doctor’s appointments and delay of therapies, resulting in emotional stress [8]. The implications of COVID-19 restrictions on the course of primary headache disorders remain largely unknown. On the one hand, such measures might lead to a worsening of headaches due to increased psychological stress and poorer healthcare resources. On the other hand, however, an improvement due to fewer work-related stressors and more self-care at home could also be a possibility.

A digital headache diary is a reliable tool to track headache attacks and potential triggers. Compared to paper-and-pencil diaries, digital documentation is associated with a higher compliance and better data quality [9]. We aimed to assess changes in headache characteristics before and during the COVID-19 lockdown period in Germany using a smartphone-based headache diary. We focused on both the immediate changes in the first 4 week after lockdown implementation and on following changes after 3 months of lockdown.

## Methods

### The app M-sense

M-sense is a commercial mobile headache application (app) available via app store for Android and iOS in Germany, Austria, and Switzerland. At the time of the data analysis, M-sense was marketed as a migraine app in a free-of-charge “Basic” version with approximately 85,000 registered users and an additional “Active” version for purchase.

Functions of M-sense comprise the documentation of headache attacks in an interactive electronic diary, along with a daily entry of predefined factors, which may influence the course of headaches. Headache features are entered according to a standardized scheme, which includes start and end of the headache, pain intensity, headache localization, headache character, presence of aura, accompanying symptoms, and acute medication use. A validated algorithm classifies single headache episodes as migraine, tension-type headache (TTH) or non-migraine / non-TTH headache [10]. A detailed description of the algorithm can be found in the publication by Roesch et al. [10]. Headache attacks are classified based on the International Classification of Headache Disorders 3 (ICHD-3) [11]. During installation, users are asked if they have already received a headache diagnosis by a healthcare professional. If this is not the case, attacks that fulfill the criteria of both TTH and probable migraine are classified as TTH. For users with a known migraine diagnosis, attacks that fulfill the criteria of probable migraine count as migraine. Headache attacks accompanied by aura or relieved by triptans qualify as migraine.

### Population and outcomes

The app developers provided us with aggregated data from all M-sense users, who entered headache-related data in this app in the four-week periods before March 22 (baseline) and after March 22 (first month of lockdown) every day. Primary outcome of this retrospective analysis was the change in monthly headache days (MHD) between baseline and the first lockdown month. Our analysis also included changes in monthly migraine days (MMD), monthly days with use of acute headache medication (AMD), mean headache and migraine intensity (on a numeric rating scale – NRS 0–10). A headache day was defined as any calendar day with a registered headache attack, regardless of the individual headache features. A migraine day was defined as each calendar day at which the user experienced a headache attack classified as migraine by M-sense. Acute headache medication consisted of triptans and non-steroidal anti-inflammatory drug (NSAID). We also assessed changes of several predefined factors including sleep duration (in 15-min intervals), self-assessed sleep quality (on a NRS 0–10), energy level (0–10), mood (0–4), stress (0–10), and level of activity (0–10).

We then performed an extension analysis of M-sense users who continued to use the app for at least 3 months after lockdown begin. In these users, we analyzed changes in the above mentioned parameters between the baseline phase, the first, second and third lockdown month.

### Statistical analysis

Statistical analysis was performed using R, version 3.6.2. For data protection, we developed the statistical code on a dummy dataset. The team of Newsenselab ran the finalized code on the real dataset. By doing so, the research team did not access personal data but received only aggregated results for the predefined endpoints. Demographics and monthly headache characteristics were summarized with descriptive statistics, using frequencies and percentages or means ± standard deviation. We compared outcomes between baseline and first month of lockdown using paired, two-tailed t-tests. In the extension analysis, outcomes were compared between all months using repeated-measurement analysis of variance (ANOVA). Post hoc pairwise comparisons were performed only if the ANOVA revealed significant results. A *p*-value ≤0.05 was considered statistically significant. *P*-values were adjusted for multiple comparisons using the Bonferroni procedure. We calculated effect sizes using Cohen’s d.

## Results

During the primary observation period, *n* = 2325 users (mean age 38.69 ± SD 11.09 years) entered data in M-sense every day. The sample consisted of *n* = 1699 females (73.1%), *n* = 271 males (11.6%), and *n* = 2 diverse sex (0.1%), while *n* = 353 (15.2%) did not provide information about sex.

Headache frequency did not show any statistical difference, with 7.01 ± 5.64 MHD during baseline and 6.89 ± 5.47 MHD in the first lockdown month (95%-CI −0.03 – 0.27, *p* = 0.999). MMD, headache and migraine intensity did also not change significantly over time (Table 1). AMD decreased slightly from 4.50 ± 3.88 before lockdown to 4.27 ± 3.81 in the first lockdown month (95%-CI 0.19–0.35, *p* < 0.001, d = 0.060).

**Table 1:** Headache characteristics and potential trigger factors during the 28-day period before and the first 28 days of the lockdown period in Germany

	Before lockdown	First lockdown month	***p*** value	95%-CI	d
**Monthly headache days (MHD)**	7.01 ± 5.64	6.89 ± 5.47	> 0.999	−0.03 – 0.27	0.022
**Monthly migraine days (MMD)**	4.98 ± 4.97	4.95 ± 4.83	> 0.999	−0.11 – 0.16	0.006
**Monthly days with acute medication use (AMD)**	4.50 ± 3.88	4.27 ± 3.81	< 0.001*	0.19–0.35	0.060
**Migraine pain intensity (NRS 0–10)**	5.30 ± 1.77	5.35 ± 1.75	> 0.999	−0.10 – 0.01	0.028
**Headache intensity (NRS 0–10)**	4.90 ± 1.71	4.92 ± 1.72	> 0.999	−0.09 – 0.01	0.011
**Activity Level (NRS 0–10)**	4.82 ± 1.56	4.76 ± 1.62	0.001*	0.03–0.09	0.038
**Energy Level (NRS 0–10)**	5.28 ± 1.42	5.34 ± 1.47	< 0.001*	−0.08 – − 0.04	0.041
**Mood (NRS 0–4)**	2.42 ± 0.53	2.45 ± 0.56	< 0.001*	−0.04 – − 0.02	0.055
**Stress level (NRS 0–10)**	3.81 ± 1.83	3.38 ± 1.86	< 0.001*	0.40–0.46	0.233
**Sleep duration (h)**	7.68 ± 0.79	7.87 ± 0.82	< 0.001*	−0.20 – − 0.17	0.236
**Sleep quality (NRS 0–10)**	5.96 ± 1.57	5.97 ± 1.61	> 0.999	−0.03 – 0.01	0.006

Values are mean ± standard deviation. *NRS* numeric rating scale, *CI* confidence interval. * = statistically significant. d = effect size, expressed as Cohen’s d.

Further analyses revealed a reduced activity level, a reduced stress level, a better mood, and an improved energy level during lockdown. Sleep duration was significantly longer, while sleep quality showed no significant change (Table 1). Effect sizes were small for all comparisons with highest values for sleep duration (d = 0.236) and stress (d = 0.233).

### Extension analysis up to 3 months after lockdown begin

In the extension analysis, we included 539 M-sense users with daily data up to the third lockdown month. These users were on average 39.07 ± 11.08 years old.

They reported 6.11 ± 5.10 MHD during baseline and 6.07 ± 5.17 MHD in the third lockdown month (*p* = 0.688 in the ANOVA between all months). MMD, AMD, migraine or pain intensity did also not show any significant change. Stress levels remained numerically below baseline in every month, but without statistical significance. Table 2 shows the monthly levels of all analyzed factors during the baseline and the first three lockdown months.

**Table 2:** Headache characteristics and potential trigger factors before lockdown begin and in the first 3 months of lockdown in Germany

	Before lockdown	First lockdown month	Second lockdown month	Third lockdown month	***p*** value^**a**^
**Monthly headache days (MHD)**	6.11 ± 5.10	5.93 ± 4.93	5.98 ± 4.83	6.07 ± 5.17	0.688
**Monthly migraine days (MMD)**	4.02 ± 4.44	4.07 ± 4.24	4.11 ± 4.21	4.23 ± 4.44	0.326
**Monthly days with acute medication use (AMD)**	4.14 ± 3.95	3.95 ± 3.88	4.17 ± 3.82	4.34 ± 4.06	0.786
**Migraine pain intensity (NRS 0–10)**	5.43 ± 1.95	5.36 ± 1.81	5.30 ± 1.91	5.29 ± 1.93	0.923
**Headache intensity (NRS 0–10)**	4.84 ± 1.86	4.86 ± 1.81	4.83 ± 1.77	4.88 ± 1.84	0.328
**Activity Level (NRS 0–10)**	4.88 ± 1.67	4.84 ± 1.70	4.90 ± 1.71	4.98 ± 1.70	0.187
**Energy Level (NRS 0–10)**	5.27 ± 1.54	5.35 ± 1.55	5.33 ± 1.57	5.33 ± 1.58	0.586
**Mood (NRS 0–4)**	2.42 ± 0.58	2.44 ± 0.58	2.46 ± 0.59	2.49 ± 0.58	0.612
**Stress level (NRS 0–10)**	3.79 ± 1.91	3.39 ± 1.95	3.59 ± 1.95	3.56 ± 1.97	0.069
**Sleep duration (h)**	7.66 ± 0.85	7,87 ± 0.89	7.73 ± 0.87	7.70 ± 0.84	0.826
**Sleep quality (NRS 0–10)**	5.97 ± 1.68	6.00 ± 1.95	6.01 ± 1.70	6.01 ± 1.68	0.159

Values are mean ± standard deviation. NRS = numeric rating scale. ^a^ *p* values of the repeated measures ANOVA between all time points.

## Discussion

In a large cohort of users of a German headache app, we observed slightly reduced self-reported stress levels during the first month of the COVID-19-related lockdown. Headache frequency and intensity remained unchanged with a small reduction of acute headache medication days in the first lockdown month. An extension analysis up to the third lockdown month revealed no significant changes in headache characteristics or lifestyle factors.

This is the first study to assess changes in headache frequency longitudinally before and after lockdown implementation using daily data from a commercial headache app. Parodi et al. evaluated through personal interviews changes in migraine severity, migraine intensity, and number of triptans per week in 49 subjects in the 2 months before and during quarantine in Italy [12]. All outcomes showed a significant improvement during lockdown. A larger Italian telephone survey also revealed a mild improvement of headache frequency and intensity as well as days with acute medication intake in 433 patients with migraine during the first lockdown month [13]. Similarly, a cohort study from the Netherlands, using a time-locked e-diary, showed a significant decrease in monthly migraine days and acute medication days during the first month of lockdown [14]. While our analysis did not detect any differences in pain frequency or intensity, the reduction of acute medication use in the first month is in line with findings from these previous studies. One possible explanation relates to the acute effects of decreased stress levels during lockdown. Changes in work and social routines might have led to a more relaxed way of living. In home office, people are usually more flexible in their time management and do not need to take acute medication immediately to “function” again. The public discussion of a potential link between NSAIDs and a negative course of COVID-19 infections could also have contributed to the slight reduction of acute medication days [15]. Of note, there was a numeric increase in the subsequent months, which might reflect habituation to the new living conditions or might also be explained by return to office / workplace with higher pressure to “function”.

All observed changes in lifestyle factors during the first lockdown month were modest and their clinical significance remains to be determined. In the extension analysis, we could not detect any significant changes anymore. This could be due to softening of the lockdown measures but could also be a result of related to the smaller sample size and thus reduced power. Importantly, our aggregated results cannot provide information on individual fates, but only an overall view of average values. While some individuals may have benefited from the lockdown measures in terms of more relax and self-care, some others might have become isolated or lost their job. A more direct influence of the disease itself through symptomatic infections is also possible, although certainly rare, as fewer than 200,000 COVID-19 cases were reported in Germany during the observation period (≈ 0.25% of the German population).

Previous research on sleep patterns during the COVID-19 pandemic is scarce: In an Indian cohort, 325 students reported an increased sleep duration during lockdown, while 203 office workers indicated reduced sleep hours [16]. Our findings are similar to the data of the Indian students. Although the differences in sleep time are small and no longer significant in the extension analysis, they are in line with reduced stress levels.

A major concern for patients with primary headache disorders in the COVID-19 pandemic may be the worsening of their condition due to reduced access to medical care [8]. However, our data does not support such hypothesis. Accordingly, a study in patients with multiple sclerosis reported improved health-related quality of life during lockdown [17]. A similar pattern may apply to headache patients, as reflected by the slightly higher levels of mood in our cohort. As the lockdown started in early spring, seasonal influences might also have contributed to mood improvement in the first lockdown month.

The reduction of physical activity due to home confinement in the pandemic has already been reported in large survey-based cohort studies [18, 19]. We detected similar reductions only in the first month of lockdown, while activity levels increased again in the subsequent months. This may be explained by the selective softening of lockdown measures (e.g. opening of gyms, increase in mobility) but also by the increased popularity of home-based exercise [20].

This study is an example on how headache data collected by an app can be used for research purposes in specific situations like the COVID-19 pandemic. Digital data collection offers the possibility of a large sample size. Nevertheless, several methodological limitations should be considered when interpreting our results. The M-sense algorithm classifies the single headache attacks, but cannot provide a final headache diagnosis. Due to the analysis of aggregated anonymous data patients could not be assessed for individually diagnosis. Previous assessments have shown that more than 70% of users have headaches compatible with the diagnosis of migraine [21]. Therefore, it is likely that our cohort consists mostly of patients with migraine but other headache disorders cannot be excluded. In addition, the app did not provide sufficient information about changes in preventive treatment or medical consultations during the observation period. Lifestyle factors were assessed using numeric rating scales, which is a simple and intuitive method, but not validated for this purpose. About 85% of M-sense users are estimated to live in Germany. The lockdown started in Austria and Switzerland (15% of users) a few days earlier than in Germany, which may have had a small impact on the data of the last baseline week. The subsequent lockdown measures were similar in all three countries. Place of residence is not collected by default in the app and subgroup analyses depending on the living place were not possible. Moreover, regional differences in continuation of lockdown measures after the first month could not be controlled for. However, considering that the majority of restrictions remained in place during the entire observation period, this is unlikely to have had relevant effects on our results. Due to the particular analysis procedure with only aggregated, anonymized data for predefined endpoints, subgroup analyses or timeline extensions were not retroactively possible. Further studies should assess which patients are more affected by lockdown measures and on which factors this depends. A longer follow-up period with the inclusion of the subsequent lockdown phases should also be considered.

## Conclusion

In conclusion, we did not observe a change in headache frequency and intensity in patients suffering from migraine and/or TTH during the first 3 months of the lockdown in Germany. A small stress reduction in the first lockdown month was not associated with an improvement in these primary headache disorders.

## Data Availability

The authors agree to share aggregated anonymized data from this analysis by request from any qualified investigator.

## Abbreviations

AMD: Monthly days with use of acute headache medication
ANOVA: Analysis of variance
App: Application
CI: Confidence interval
COVID-19: Coronavirus disease 2019
MHD: Monthly headache days
MMD: Monthly migraine days
NSAID: Non-steroidal anti-inflammatory drug
SE: Standard error
TTH: Tension-type headache
NRS: Numeric rating scale
WHO: World Health Organization
